# Is there a relationship between opioid use and transient global amnesia?

**DOI:** 10.1111/ene.16134

**Published:** 2023-11-13

**Authors:** Jed A. Barash, Megha Parikh, Rosa Ergas, Alfred DeMaria

**Affiliations:** ^1^ Veterans' Home Chelsea Massachusetts USA; ^2^ Massachusetts Department of Public Health Boston Massachusetts USA

**Keywords:** amnestic syndrome, opioid, transient global amnesia

## Abstract

**Background and purpose:**

Opioid‐associated amnestic syndrome (OAS) and transient global amnesia (TGA) are conditions with clinical overlap. We therefore sought to determine whether opioid use might be associated with TGA.

**Methods:**

Data from the Massachusetts Department of Public Health Syndromic Surveillance program were queried to ascertain the frequency of opioid use among emergency department (ED) encounters for TGA compared to that for all other ED visits between January 2019 and June 2023.

**Results:**

A total of 13,188,630 ED visits were identified during the study period. Of 1417 visits for TGA, one visit met the exposure definition for opioid use. There were 13,187,213 visits for other indications, 57,638 of which were considered opioid‐exposed. The odds ratio for the relationship between opioid use and TGA was 0.16 (95% confidence interval 0.02, 1.14).

**Conclusion:**

Despite the clinical overlap between OAS and TGA, surveillance data from ED visits in Massachusetts do not suggest that opioid use is a risk factor for TGA, indicating that OAS and TGA are distinct entities.

## INTRODUCTION

Transient global amnesia (TGA) is an idiopathic phenomenon, characterized by isolated anterograde memory loss that resolves within 24 h [1]. The condition has frequently been associated with punctate lesions of the hippocampus on diffusion‐weighted magnetic resonance imaging (MRI). One retrospective, observational study demonstrated these hippocampal abnormalities in approximately 70% of 390 consecutive TGA patients overall and bilaterally in over 20% [2]. Among potential risk factors proposed for TGA are general anesthetics and drugs of abuse [3, 4]. The recent emergence, therefore, of a rare, acute‐onset anterograde amnestic syndrome occurring in the setting of opioid use, closely linked to fentanyl, is of special interest [5, 6, 7]. This opioid‐associated amnestic syndrome (OAS) is characterized by diffuse lesions of the hippocampus bilaterally on diffusion‐weighted MRI. Reports indicate that OAS lasts for weeks to months and in some instances, a year or longer [8].

Opioid‐associated amnestic syndrome can be easily distinguished from TGA when there is an impaired level of consciousness or sufficient follow‐up observation. However, OAS cases may present with similar features to those of TGA, including frequent repetition, and absence of altered levels of consciousness [5]. Moreover, the possibility of “transient” OAS cases of shorter duration (and potentially attributed to TGA) could be considered.

In this context, we sought to determine whether opioid use might be associated with TGA. Thus, data from the Massachusetts Department of Public Health Syndromic Surveillance (MDPH SyS) program—established to facilitate collection of emergency department (ED) visit data for public health monitoring—were queried to ascertain the frequency of opioid use among ED encounters for TGA compared to that for all other ED visits.

## METHODS

### Syndromic surveillance

Data in MDPH SyS comprise free text and the coded reason(s) for an ED encounter, including patient discharge diagnostic codes and demographics [9]. ED visit data from January 2019 (the first calendar year in which all Massachusetts EDs were fully enrolled into the MDPH SyS) to June 2023 (the last month of data available during the study period) were considered. Queries centered on two definitions of interest: opioid use (exposure) and TGA (outcome). To determine the relative odds of TGA occurring given an opioid exposure, an odds ratio with a confidence interval (CI) was calculated based on ED visits for TGA, those for all other indications, and documented opioid use.

### Exposure definition: Opioid use

The algorithm used to identify opioid exposure was modified from the opioid overdose definition (version 3) created by partners in the Centers for Disease Control and Prevention's (CDC) National Center for Injury Prevention and Control and incorporated in the National Syndromic Surveillance Program's (NSSP) Early Notification of Community‐Based Epidemics (ESSENCE) web‐based analytical tool [10]. This modification extends the definition to encompass ED visits related to opioid use, abuse, and dependence with intoxication as well as new International Classification of Diseases, Tenth Revision, Clinical Modification (ICD‐10‐CM) codes for poisoning by fentanyl and fentanyl analogs in addition to other synthetic narcotics. While the exposure definition is intended to capture encounters for acute intoxication and poisoning, it does not further differentiate recreational or prescription usage. Moreover, the dataset does not include toxicology results for confirmation.

### Outcome definition: Transient global amnesia

The TGA definition algorithm searched chief complaint text and diagnostic codes for terms and codes associated with TGA. The term “transient global amnesia” and the ICD‐10‐CM code G 45.4 code (TGA) were used.

The data for this study were deidentified and collected as a component of public health surveillance and the NSSP, the latter a partnership between the CDC, state and local health departments, as well as healthcare facilities. The compilation and analysis of these data are considered part of public health surveillance and not human subject research; thus, for secondary analyses, institutional review board approval and informed consent are not required.

## RESULTS

A total of 13,188,630 ED visits were identified during the study period (Table 1). Of 1417 visits for TGA, one visit met the exposure definition for opioid use. There were 13,187,213 visits for other indications, 57,638 of which were considered opioid‐exposed. The odds ratio for the relationship between opioid use and TGA was 0.16 (95% CI 0.02, 1.14).

**TABLE 1:** Surveillance case counts of opioid use among emergency department (ED) visits for transient global amnesia compared to that for all other ED visits in Massachusetts, January 2019 to June 2023.

	TGA	No TGA	Total
Opioid use	1	57,638	57,639
No opioid use	1416	13,129,575	13,130,991
Total	1417	13,187,213	13,188,630

Abbreviation: TGA, transient global amnesia.

## DISCUSSION

Despite the clinical overlap between OAS and TGA, surveillance data from ED visits in Massachusetts between January 2019 and June 2023 do not suggest that opioid use is a risk factor for TGA. Proposed mechanistic differences between OAS and TGA might begin to offer insight into this observation. Although OAS is thought to result primarily from an excitotoxic effect of opioids on the hippocampus [11], the leading causes posited for TGA are vascular or migrainous in nature, including ischemia and cortical spreading depression, respectively [1]. Additionally, patients with OAS commonly present with altered consciousness due to respiratory depression [5], whereas those with TGA do not [1].

Several limitations of this study are worth considering. First, it included only ED data. However, the acute nature of TGA generally prompts presentation to the ED rather than other clinical settings, suggesting that potential cases were well captured. Secondly, cases were not validated with medical record review. Nevertheless, based on the 2020 census for Massachusetts (population ~7 million) and ED surveillance data during the study period, the average annual incidence of TGA would be approximately 4.5 per 100,000, a rate near that reported previously in the United States [12]. Even allowing for overdiagnosis, reversal of these findings to a positive association would be unlikely. Finally, while ED visits do not represent unique patients, the transient nature of TGA and relative infrequency of its recurrence [1] indicate that this factor had minimal, if any, impact on the outcome of the study. In spite of these barriers, the data here therefore suggest that OAS and TGA are indeed distinct entities.

## AUTHOR CONTRIBUTIONS

**Jed A. Barash**: Conceptualization; investigation; writing—original draft; methodology; writing—review and editing. **Megha Parikh**: Investigation; writing—review and editing; methodology. **Rosa Ergas**: Investigation; methodology; writing—review and editing. **Alfred DeMaria Jr.**: Investigation; conceptualization; writing—review and editing; methodology.

## FUNDING INFORMATION

This work was funded by the Centers for Disease Control and Prevention grant number NU17CE925012.

## CONFLICT OF INTEREST STATEMENT

The authors declare no conflicts of interest.

## Data Availability

The data for this study were deidentified and collected as a component of public health surveillance and the National Syndromic Surveillance Program, the latter a partnership between the Centers for Disease Control and Prevention, state and local health departments, as well as health care facilities.
